# Optimizing Tuberculosis Case Detection through a Novel Diagnostic Device Placement Model: The Case of Uganda

**DOI:** 10.1371/journal.pone.0122574

**Published:** 2015-04-01

**Authors:** Mai T. Pho, Sarang Deo, Kara M. Palamountain, Moses Lutaakome Joloba, Francis Bajunirwe, Achilles Katamba

**Affiliations:** 1 Department of Medicine, Sections of Hospital Medicine and Infectious Diseases & Global Health, University of Chicago, Chicago, United States of America; 2 Indian School of Business, Hyderabad, India; 3 Kellogg School of Management, Northwestern University, Evanston, United States of America; 4 Department of Medical Microbiology, Makerere University College of Health Sciences, Kampala, Uganda; 5 Department of Community Health, Mbarara University of Science and Technology, Mbarara, Uganda; 6 Department of Medicine, Makerere University College of Health Sciences, Kampala, Uganda; University of Delhi, INDIA

## Abstract

**Background:**

Xpert MTB/RIF (Xpert) is being widely adopted in high TB burden countries. Analysis is needed to guide the placement of devices within health systems to optimize the tuberculosis (TB) case detection rate (CDR).

**Methods:**

We used epidemiological and operational data from Uganda (139 sites serving 87,600 individuals tested for TB) to perform a model-based comparison of the following placement strategies for Xpert devices: 1) Health center level (sites ranked by size from national referral hospitals to health care level III centers), 2) Smear volume (sites ranked from highest to lowest volume of smear microscopy testing), 3) Antiretroviral therapy (ART) volume (sites ranked from greatest to least patients on ART), 4) External equality assessment (EQA) performance (sites ranked from worst to best smear microscopy performance) and 5) TB prevalence (sites ranked from highest to lowest). We compared two clinical algorithms, one where Xpert was used only for smear microscopy negative samples versus another replacing smear microscopy. The primary outcome was TB CDR; secondary outcomes were detection of multi-drug resistant TB, number of sites requiring device placement to achieve specified rollout coverage, and cost.

**Results:**

Placement strategies that prioritized sites with higher TB prevalence maximized CDR, with an incremental rate of 6.2–12.6% compared to status quo (microscopy alone). Diagnosis of MDR-TB was greatest in the TB Prevalence strategy when Xpert was used in place of smear microscopy. While initial implementation costs were lowest in the Smear Volume strategy, cost per additional TB case detected was lowest in the TB prevalence strategy.

**Conclusion:**

In Uganda, placement of Xpert devices in sites with high TB prevalence yielded the highest TB CDR at the lowest cost per additional case diagnosed. These results represent novel use of program level data to inform the optimal placement of new technology in resource-constrained settings.

## Introduction

Tuberculosis (TB) remains a major global public health challenge, causing substantial morbidity and mortality [1]. One of the most important risk factors for the increasing TB burden is HIV/AIDS, which contributes to the difficulty in diagnosing TB in co-infected patients [1]. The introduction of the Xpert MTB/RIF (Cepheid, Sunnyvale, CA, USA) represents the first true “game-changer” in the field of TB diagnostics in decades due to improved sensitivity, ease of use, and rapid turn-around-time of results made possible by the molecular platform, and in 2010, the World Health Organization endorsed the use of the Xpert device [2, 3].

The feasibility and cost-effectiveness of the Xpert have been well studied and support the implementation of the device in resource-limited settings [4–8]. When allocating new diagnostics, it has been recommended that public health decision-makers utilize not only data from such technical studies and registration trials, but also examine the existing epidemiology, health care infrastructure and clinical practice to optimize implementation and scale up [9]. However, few evidence-based guidelines exist on how the new technology should be integrated into a country’s existing laboratory infrastructure, or how placement of the new technology should be prioritized [10].

In Uganda, a country with high TB and HIV prevalence and the focus of our analysis, Xpert testing is provided mainly to a limited number of individuals with known HIV infection. To guide the placement of the Xpert device within the existing national tuberculosis laboratory network, priority is given to centers with high smear microscopy volumes, centers providing HIV/AIDS care, and sites serving areas with traditionally poor health care access, including islands and prisons [11]. These criteria have led to placement of Xpert devices in regional referral hospitals, district hospitals and some large volume HIV/AIDS care centers.

While prioritization of Xpert placement in sites with high smear volume and HIV and TB prevalence is intuitive given the burden of TB disease at these locations, it remains unclear as to which of these criteria will maximize case detection rates for TB. Further, operational factors such as the quality of smear microscopy may also impact optimal integration of the Xpert device, as sites with poorer microscopy performance are likely to benefit more from this device. In this analysis we used national epidemiologic and program level data to perform a model-based comparison of the impact of different placement strategies for the Xpert device on the TB case detection rate (CDR) and cost in Uganda. To our knowledge this is the first attempt to use health facility level operational data to evaluate the placement of the Xpert device.

## Methods

**Fig 1:**
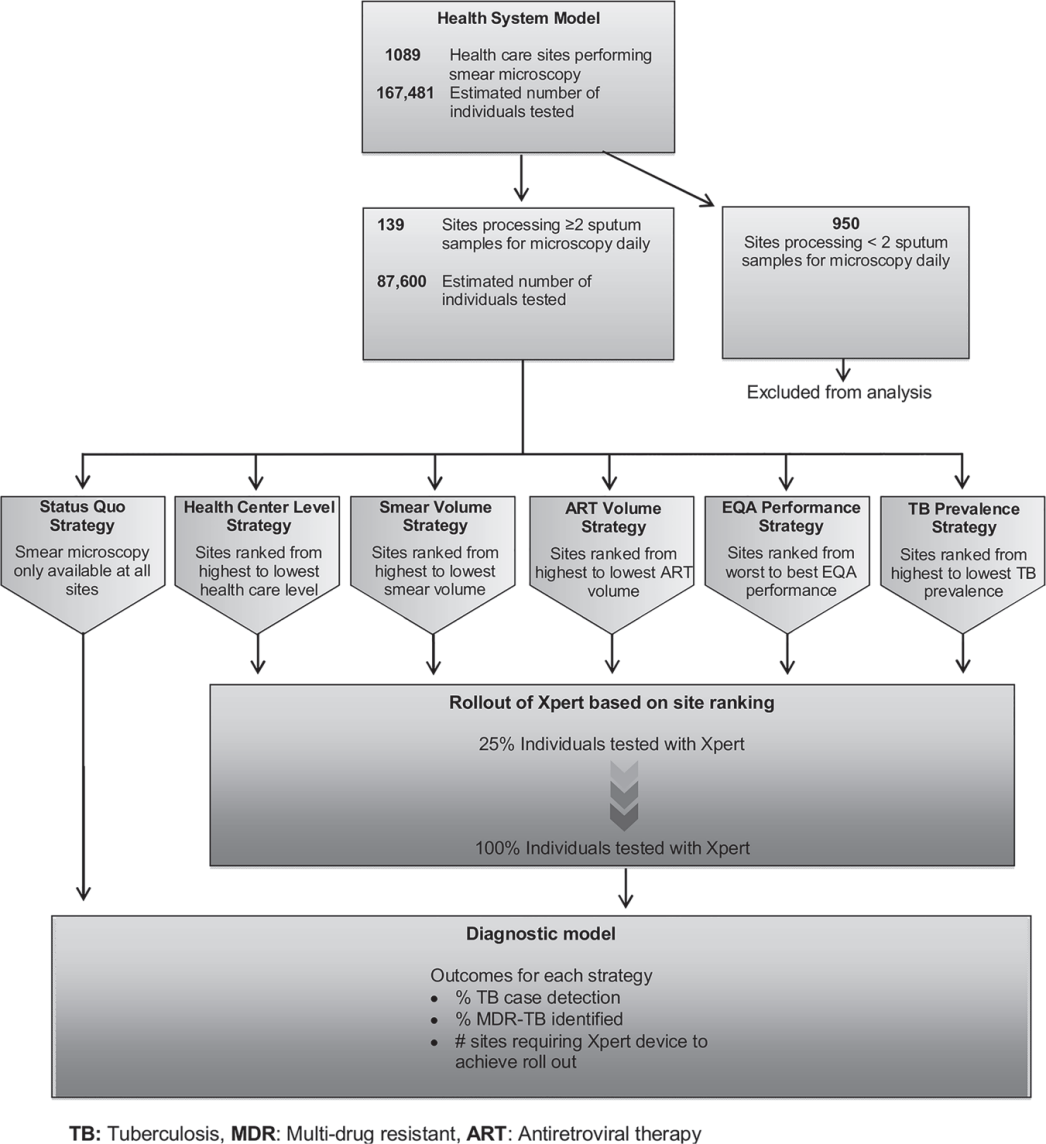
Analytical schema. Of the total 1089 health care sites with smear available in Uganda, 139 met inclusion criteria (performed on average at least two samples daily) for analysis. These 139 sites were variably ranked based on placement schema as indicated in pentagonal boxes. Sensitivity and specificity of smear for all sites was adjusted by EQA data. Xpert was rolled out over the patient population. The decision tree was used to calculate estimates for TB case detection rates, number of MDR-TB cases, and number of health care sites that would require Xpert device placement to achieve Xpert rollout by any given placement schema.

We developed a decision-analytic model using TreeAge Pro 2012 (Williamstown, MA) and Microsoft Excel 2010 (Redmond, WA). The analytic schema is presented in Fig. 1. The primary outcome was TB CDR. Secondary outcomes were cases of MDR-TB identified, number of sites requiring Xpert devices and cost.

### Ethics

The study was approved by the Makerere University School of Medicine Research and Ethics Committee and the Ugandan National Council of Science and Technology. All records were de-identified of protected health information prior to analysis.

### Estimation of Model Parameters

We created a model of all 1,089 sites in the Uganda healthcare system that provided smear microscopy services for the diagnosis of tuberculosis from January 1 to December 31, 2011. Site level data were obtained from the National TB Reference Laboratory (NTRL), the National TB Control Program, and the National AIDS Control Program and are provided in S1 Dataset (supporting information) [12–14]. Individual sites were characterized by level of health care provided at the facility, TB prevalence, annual smear microscopy volume, cumulative number of patients enrolled in antiretroviral therapy, and quality of microscopy performance (sensitivity and specificity) based on external quality assessment (EQA) performed by the NTRL through expert review of a sample of smears sent by each site. Exclusion criteria included sites processing less than two sputum samples for smear microscopy daily (Fig. 1). Number of individuals tested per site was estimated by dividing the total annual smear samples reported to the NTRL in 2011 at each site by 1.5 to reflect the average number of samples provided per individual based on expert opinion. Prevalence of TB at smear microscopy centers was estimated by obtaining smear microscopy results as reported to the NTRL and adjusting them by the EQA-derived sensitivity and specificity for each facility as described in further detail below. Rates of multi-drug resistant disease and HIV co-infection were based on a national drug resistance survey of TB which examined a nationally representative sample of new and previously treated sputum smear-positive TB patients utilizing Lowenstein-Jensen (L-J) culture methods, PCR confirmation of Mycobacterium tuberculosis, and drug susceptibility testing using the L-J proportional method [1, 15]. The HIV infection rate among individuals evaluated at sites that were known to be ART centers was assumed to be 100% HIV.

### Diagnostic model structure and validation

The diagnostic algorithm in the model varied depending on whether Xpert was placed at a site or not. In sites where Xpert was not placed, individuals were evaluated using two sputum samples for smear microscopy using the Ziehl-Neelsen staining procedure. Smear negative individuals underwent clinical evaluation, with sensitivity and specificity for TB diagnosis based on published literature. In sites where Xpert was placed, individuals were first examined using two sputum samples for microscopy. Sputum samples for smear negative individuals were examined using the Xpert device including rifampicin resistance testing (integrated diagnostic algorithm). AFB culture and drug susceptibility testing was not considered in the model as access to these tests is limited to only 0.6 per 5 million individuals in the general population via line probe assay in Uganda.

The model was externally validated to WHO estimations of TB case detection in 2011 for Uganda, prior to the introduction of Xpert into the national laboratory.

### Xpert Placement Strategies

We examined five different Xpert placement strategies based on the current recommendations of the Ugandan National TB Reference Laboratory, as well as novel strategies based on the microscopy performance of individual sites and site-specific TB prevalence (Fig. 1).

1. **Health Center Level.** In the Health Center Level strategy, Xpert was allocated to health care sites based on the status of the site in the hierarchy of facilities as follows. Xpert was placed first in national referral hospitals, followed by regional referral hospitals, general hospitals, health care level IV sites (considered mini-hospitals, capable of admitting patients, with laboratory and staffed by physicians) and health care level III sites (typically outpatient clinic and maternity ward, with laboratory and staffed by clinical officer). Within each level, sites were prioritized by highest to lowest smear microscopy volume.
2. **Smear Volume.** In the Smear Volume strategy, Xpert was placed in sites ranked by highest to lowest annual number of smear samples evaluated by microscopy.
3. **ART Volume.** In the Antiretroviral (ART) Volume strategy, Xpert was allocated to health care sites ranked from highest to lowest cumulative number of patients ever enrolled in ART and subsequently by smear volume, if additional devices were available after allocation to ART sites. Based on current expectations of stakeholders in the Ugandan Ministry of Health, Xpert was assumed to be placed in the site laboratories, as opposed to patient care areas, thus allowing access to for both HIV-infected and non-infected patients.
4. **EQA Performance.** In the External Quality Assessment (EQA) Performance strategy, Xpert was placed in sites ranked by lowest to highest sensitivity and specificity of smear microscopy by each site according to EQA. EQA was conducted by the NTRL on random samples submitted by each site for retesting and comparison of observed results by the site compared to rechecked results by the reference laboratory.
5. **TB Prevalence.** In the TB Prevalence strategy, Xpert was placed in sites ranked from highest to lowest TB prevalence. As noted above, the prevalence of TB at each site was estimated by obtaining smear microscopy results as reported to the NTRL and adjusting them by the EQA performance of the site.

For each strategy, increasing Xpert rollout (0%, 25%, 50%, 75%, 100%) to the total patient population tested for TB was considered, starting with the highest ranked sites and proceeding to the lowest ranked sites. In any given strategy, individuals without access to Xpert due to incomplete rollout were evaluated as in the status quo, for which only smear microscopy and clinical evaluation were available.

### Test characteristics

Baseline sensitivity and specificity of smear microscopy, clinical examination, and Xpert MTB/RIF were obtained from demonstration studies in the literature [5, 8]. Site-specific smear microscopy performance was calculated by multiplying the sensitivity and specificity derived via routine EQA and the published sensitivity and specificity.

### Sensitivity Analysis

Several sensitivity analyses were performed to assess the robustness of the results. We assessed the impact of switching the diagnostic algorithm to reflect the current algorithm adopted in South Africa, where Xpert is performed instead of smear microscopy as opposed to only used on smear microscopy negative samples [16]. We also varied EQA-derived sensitivity and specificity at each site within +10% to -10% of the base case to reflect the limitations of the EQA process in assessing the quality of smear microscopy at the sites.

### Cost analysis

The implementation cost of providing Xpert in the first year of rollout was estimated based on the five year amortization of a four-bay Xpert device, assuming a capital outlay for device of $17,000 2014 USD, a device life of 5 years, and discount rate of 3% [17]. Annual costs attributed to each strategy were calculated adding test costs (the number of tests performed multiplied by the cost per test ($9.98 2014 USD)) and device costs (the number of devices required based on sites with Xpert available multiplied by the cost of the device as above) [17]. Cost per additional TB cases detected was calculated by dividing incremental case detection for each strategy over one year compared to the use of smear microscopy alone. Costs of personnel, training, and infrastructural requirement were not available and hence excluded in the analysis.

## Results

### Model Cohort and base case results

Of the total of 1089 sites performing smear microscopy in 2011, 139 sites met the inclusion criteria of processing an average of at least two sputum samples daily (Table 1). 87,600 individuals evaluated for TB at these sites were simulated in the diagnostic model. This represented 52% of all estimated individuals tested in 2011. The mean TB prevalence was 25.3%. After adjusting for EQA, the average sensitivity of smear microscopy in HIV-negative and HIV-positive individuals was 0.654 and 0.404, respectively (Table 2). Table 1 describes the characteristics of individuals served at each health center level, including mean number, number enrolled on ART, TB prevalence, as well as the sensitivity and specificity of smear microscopy performed at these sites.

**Table 1:** Characteristics of sites included in analysis.

	Value	Source
Total sites selected in analysis	139	[12]
Smear volume for selected sites, 2011	131,400	[12]
Estimated number individuals tested for TB	87,600	[12]
Cumulative patients enrolled in ART	149,633	[14]
Health center level, number of sites (%)		[12]
National referral hospital	4 (2.1%)	[12]
Individuals tested for TB, mean	1928	[12]
ART Volume, mean	1699	[14]
Microscopy sensitivity by EQA, mean	0.89	[13]
Microscopy specificity by EQA, mean	0.95	[13]
TB prevalence, mean	0.36	[12]
Regional referral hospital	11 (8.0%)	[12]
Individuals tested for TB, mean	924	[12]
ART Volume, mean	2925	[14]
Microscopy sensitivity by EQA, mean	0.85	[13]
Microscopy specificity by EQA, mean	0.99	[13]
TB prevalence, mean	0.29	[12]
High volume HIV center	12 (8.6%)	[12]
Individuals tested for TB, mean	577	[12]
ART Volume, mean	2360	[14]
Microscopy sensitivity by EQA, mean	0.85	[13]
Microscopy specificity by EQA, mean	0.98	[13]
TB prevalence, mean	0.24	[12]
Hospital	52 (37.4%)	[12]
Individuals tested for TB, mean	652	[12]
ART Volume, mean	1180	[14]
Microscopy sensitivity by EQA, mean	0.92	[13]
Microscopy specificity by EQA, mean	0.98	[13]
TB prevalence, mean	0.24	[12]
Health center level IV	37 (26.6%)	[12]
Individuals tested for TB, mean	504	[12]
ART Volume, mean	481	[14]
Microscopy sensitivity by EQA, mean	0.93	[13]
Microscopy specificity by EQA, mean	0.98	[13]
TB prevalence, mean	0.22	[12]
Health center level III	23 (16.5%)	[12]
Individuals tested for TB, mean	445	[12]
ART Volume, mean	447	[14]
Microscopy sensitivity by EQA, mean	0.88	[13]
Microscopy specificity by EQA, mean	0.98	[13]
TB prevalence, mean	0.24	[12]

TB: Tuberculosis, ART: Antiretroviral therapy, EQA: External quality assurance

**Table 2:** Demographics of patient population included in analysis and model input parameters.

Parameter	Base Case Value	Reference
**Cohort Characteristics**		
Prevalence of TB amongst tested individuals. mean	0.253	[12]
Smear-positive TB among HIV-negative TB cases	0.723	[5, 8]
Smear-positive TB among HIV-positive TB cases	0.446	[5, 8]
Treatment-experience amongst TB cases	0.1	[1]
MDR prevalence amongst treatment-naïve, TB cases	.011	[1, 8]
MDR prevalence amongst treatment-experienced TB cases	0.12	[1, 8]
HIV prevalence amongst TB cases	0.53	[1]
**Diagnostic Parameters**		
*Sensitivity*		
Smear microscopy, HIV-negative	0.654	[5, 8, 13]
Smear microscopy, HIV-positive	0.404	[5, 8, 13]
Xpert MTB/RIF, smear-positive TB cases	0.983	[5, 8]
Xpert MTB/RIF, smear-negative, HIV-negative cases	0.793	[5, 8]
Xpert MTB/RIF, smear-negative, HIV-positive cases	0.718	[5, 8]
Xpert MTB/RIF rifampin testing	0.983	[5, 8]
Clinical diagnosis of TB	0.444	[5, 8]
*Specificity*		
Smear microscopy	0.982	[5, 8, 13]
Xpert MTB/RIF	0.990	[5, 8]
Xpert MTB/RIF rifampin testing	0.983	[5, 8]
Clinical diagnosis of TB	0.869	[5, 8]
*Return for results*, *probability (range)*		
While awaiting smear microscopy	1 (.87–1)	[18]
While awaiting for Xpert MTB/RIF	1 (.74–1)	[5, 19]

TB: Tuberculosis, MDR: Multi-drug resistant, ART: Antiretroviral therapy. EQA: External quality assurance

**Fig 2:**
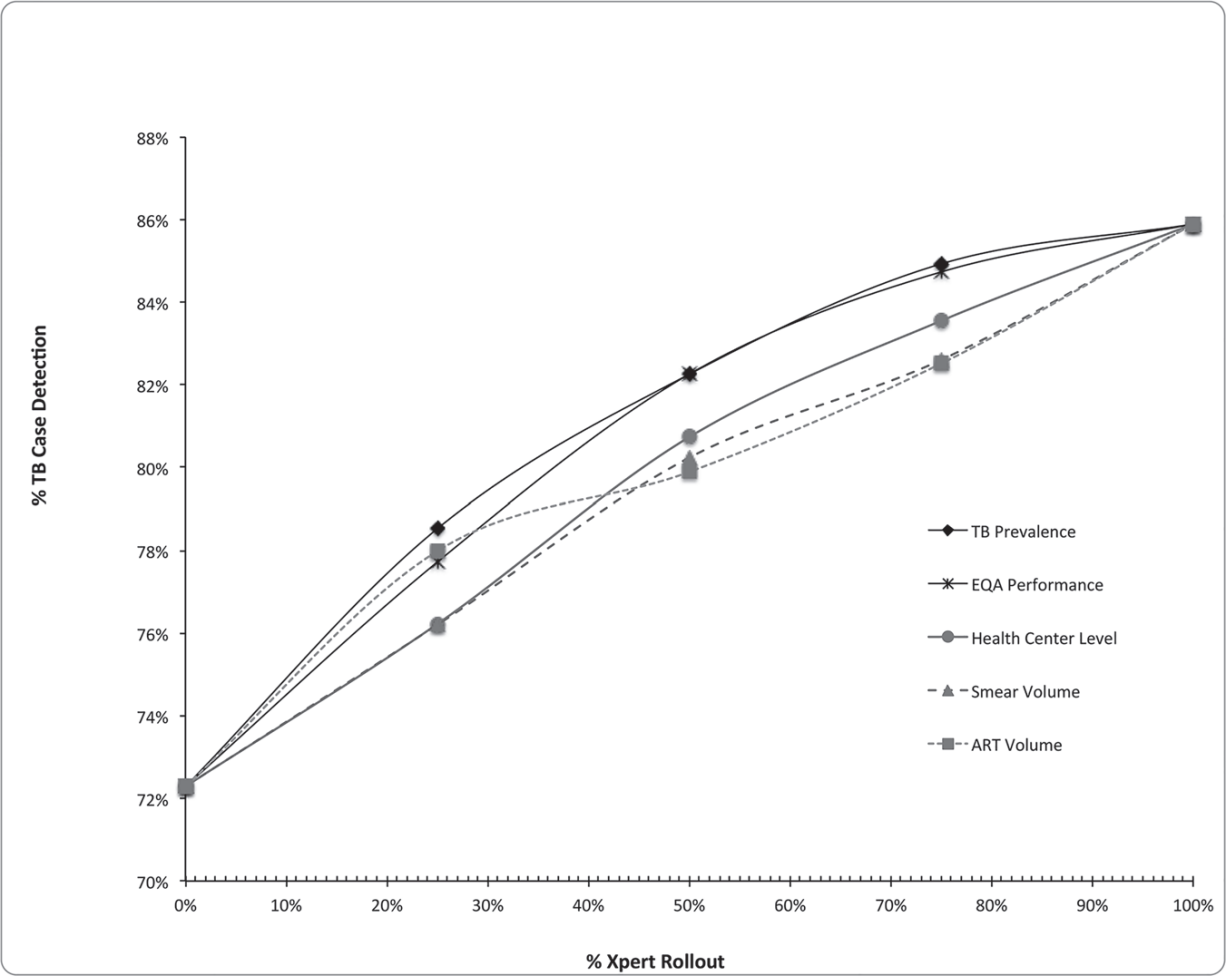
TB CDR by placement strategy using integrated diagnostic algorithm. This figure demonstrates the TB case detection rates by Xpert placement strategy as % of individuals with access to Xpert increases. Xpert is used for smear microscopy negative individuals only. Case detection rate is defined by # TB cases diagnosed / estimated total TB cases.

TB case detection rate in the status quo (diagnosis by smear microscopy only) was estimated to be 72.3% (Table 3). This rate lay within the range of WHO estimates of 51–76% [1]. Given the lack of AFB culture and drug susceptibility testing, no cases of MDR-TB were identified in the status quo. Xpert placement in sites prioritized by highest to lowest TB prevalence led to a greatest number of TB diagnoses among all strategies. Compared to the status quo, incremental case detection rates increased from 6.2–12.6% in the TB Prevalence strategy as Xpert was made available to 25–75% of the patient population (Table 3). ART volume strategy was second best for coverage of less than 30% but its performance became inferior to that of EQA performance strategy for coverage greater than 30% (Fig. 2). Case detection rates were similar in the Health Center Level and Smear Volume strategies.

**Table 3:** Base case results.

Placement Strategy		**% of individuals with access to Xpert		
**Status Quo, smear only**		**0%	**0%	**0%
	**Case Detection**	**72.3%**	**72.3%**	**72.3%**
**Health Center Level**		**25%	**50%	**75%
	**Case Detection**	**76.2%**	**80.7%**	**83.6%**
	% MDR TB detected	11.4%	19.9%	27.2%
	Number of sites with Xpert	17	44	87
**Smear Volume**		**25%	**50%	**75%
	**Case Detection**	**76.2%**	**80.2%**	**82.6%**
	% MDR TB detected	11.4%	19.7%	26.2%
	Number of sites with Xpert	14	39	79
**ART Volume**		**25%	**50%	**75%
	**Case Detection**	**78.0%**	**79.9%**	**82.5%**
	% MDR TB detected	13.5%	20.9%	28.0%
	Number of sites with Xpert	39	72	104
**EQA Performance**		**25%	**50%	**75%
	**Case Detection**	**77.7%**	**82.3%**	**84.7%**
	% MDR TB detected	9.3%	18.9%	24.4%
	Number of sites with Xpert	34	59	86
**TB Prevalence**		**25%	**50%	**75%
	**Case Detection**	**78.5%**	**82.3%**	**84.9%**
	% MDR TB detected	14.0%	23.4%	29.9%
	Number of sites with Xpert	28	57	96

TB case detection by strategy and increasing access to Xpert.

** Indicates percent of the patient population with access to the Xpert device

**Fig 3:**
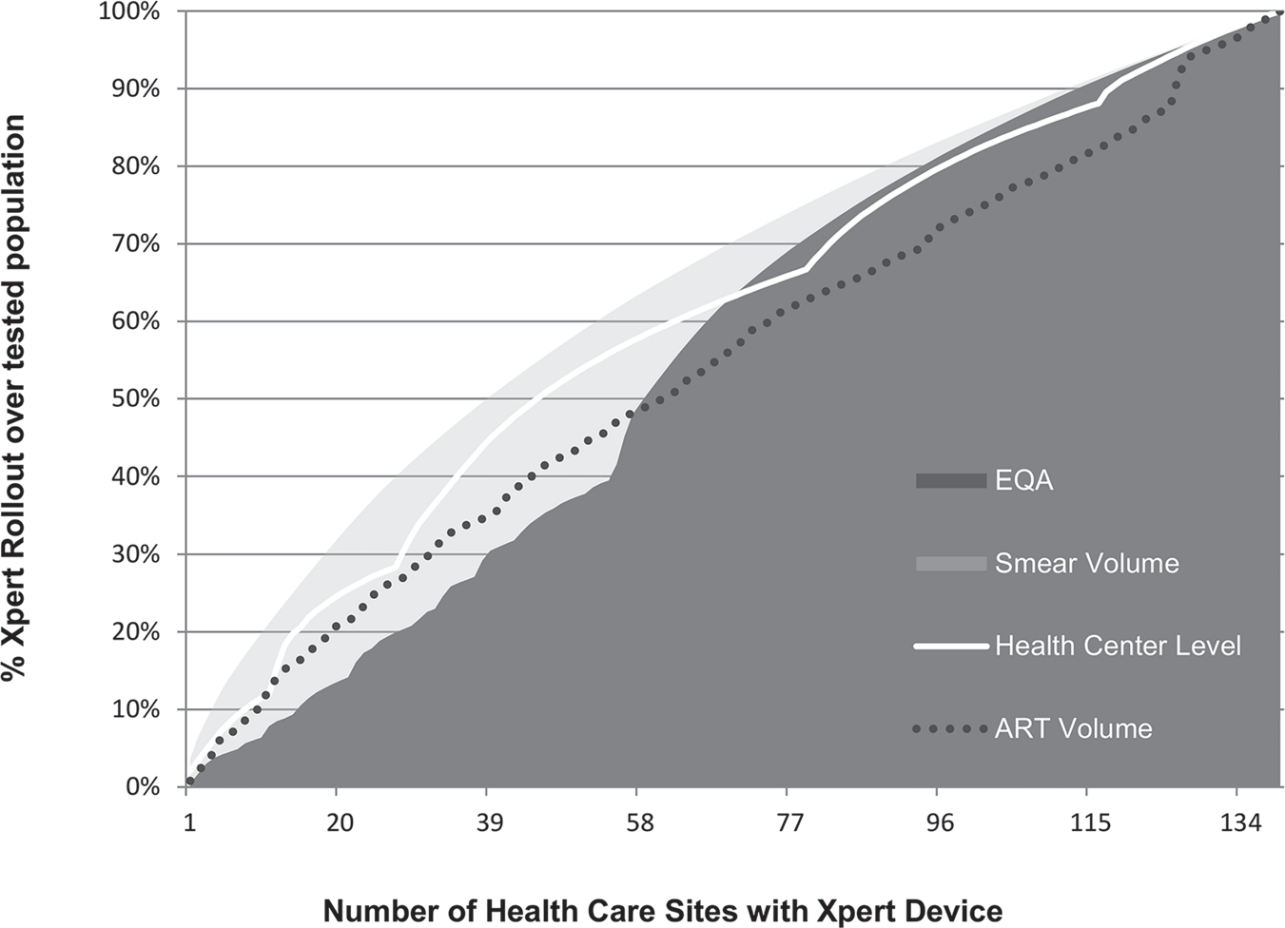
Number of health care sites with Xpert by placement strategy. This figure demonstrates the number of health care sites with Xpert device placement as the % of individuals with access to Xpert increases. Variation by strategy reflects different volumes of individuals tested at each site, and different sites selected based on placement strategy.

The number of Xpert devices required for the ART Volume strategy was greater at any given level of Xpert rollout as compared to other strategies (Table 3). For example, to achieve testing of 25% of the patient population with Xpert, 39 sites required Xpert placement in the ART Volume strategy as compared to 17 sites in the Health Center Level strategy and 14 sites in the Smear Volume strategy (Table 3, Fig. 3). This variation in the number of sites reflects the differences in the smear volume of sites prioritized based on placement strategies. In the above example, the Smear Volume strategy prioritized the highest volume smear microscopy sites, resulting in fewer numbers of sites requiring Xpert placement at assigned rollout, whereas the ART Volume strategy prioritized sites based on cumulative ART enrollment, which performed fewer smears per site in comparison. Diagnosis of MDR-TB was greatest in the TB Prevalence strategy (Table 3), with an increase of 0.5–2.5% greater MDR-TB detection compared to the next best strategy.

### Sensitivity Analysis

**Fig 4:**
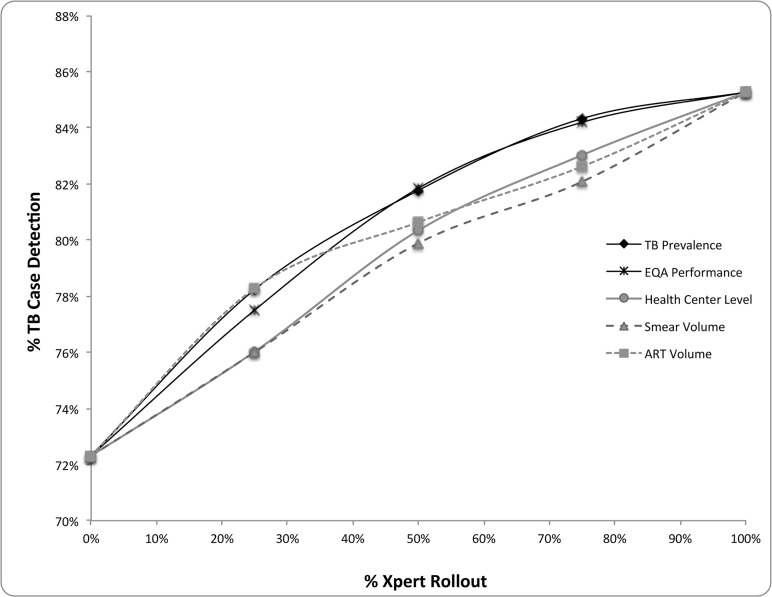
TB CDR by placement strategy when Xpert replaces smear microscopy. This figure demonstrates the TB case detection rates by Xpert placement strategy as % of individuals with access to Xpert increases. Xpert completely replaces smear microscopy in the diagnostic algorithm. Case detection rate is defined by # TB cases diagnosed / estimated total TB cases

When the diagnostics algorithm was adjusted to completely replace smear microscopy with the Xpert device (versus integrating the Xpert with smear microscopy for smear-negative individuals only), the ART Volume strategy was superior in both total CDR and detection of MDR-TB to the TB prevalence strategy when Xpert availability was limited to less than 39% of the patient population. At higher levels of device availability the TB prevalence strategy became superior (Table 4, Fig. 4).

**Table 4:** Sensitivity analysis based on use of Xpert only (no smear microscopy) where Xpert device is available.

Placement Schema		**% of individuals with access to Xpert		
**Status Quo, smear only**		0%	0%	0%
	**Case Detection**	**72.3%**	**72.3%**	**72.3%**
**Health Center Level**		**25%	**50%	**75%
	**Case Detection**	**76.0%**	**80.3%**	**83.0%**
	% MDR TB detected	26.7%	50.5%	67.5%
	Number of sites with Xpert	17	44	87
**Smear Volume**		**25%	**50%	**75%
	**Case Detection**	**76.0%**	**79.9%**	**82.1%**
	% MDR TB detected	25.8%	49.3%	63.6%
	Number of sites with Xpert	14	39	79
**ART Volume**		**25%	**50%	**75%
	**Case Detection**	**78.3%**	**80.6%**	**82.6%**
	% MDR TB detected	39.3%	55.5%	68.1%
	Number of sites with Xpert	39	72	104
**EQA Performance**		**25%	**50%	**75%
	**Case Detection**	**75.5%**	**81.8%**	**84.2%**
	% MDR TB detected	25.3%	52.0%	68.3%
	Number of sites with Xpert	34	59	86
**TB Prevalence**		**25%	**50%	**75%
	**Case Detection**	**78.2%**	**81.8%**	**84.3%**
	% MDR TB detected	35.3%	58.1%	75.5%
	Number of sites with Xpert	28	57	96

** Indicates percent of the patient population with access to the Xpert device

When EQA-derived sensitivity and specificity was varied to underestimate the observed microscopy sensitivity and specificity by 10%, base case results remained stable. However, the superiority of placement by the EQA Performance strategy compared to placement by ART Volume was lost when EQA—derived sensitivity and specificity was varied to overestimate observed microscopy performance by greater than 2%. (Results not shown)

### Costs

Implementation costs in the first year of Xpert device rollout were greatest in the ART volume strategy and were lowest in the Smear Volume strategy (Table 5). This finding was due to the greater number of sites requiring Xpert device placement at each stage of rollout in the ART volume strategy compared to the remaining strategies. Based on the measure of incremental cost per additional tuberculosis case diagnosed (compared to smear microscopy alone), the ART volume strategy remained most costly, whereas TB prevalence strategy was least costly (Table 5).

**Table 5:** Implementation cost of Xpert in the first year of Xpert rollout and cost per additional TB case diagnosed by strategy.

	Implementation cost in the first year			Cost per additional TB case diagnosed compared to status quo		
Placement Schema	**25%	**50%	**75%	**25%	**50%	**75%
Health Center Level	$235,392	$523,916	$874,923	$193	$236	$307
Smear Volume	$229,984	$504,829	$855,255	$189	$239	$324
ART Volume	$370,689	$672,008	$950,370	$230	$330	$363
EQA Performance	$299,932	$577,209	$867,219	$193	$225	$279
TB Prevalence	$269,492	$558,494	$897,277	$155	$218	$285

** Indicates percent of the patient population with access to the Xpert device

## Discussion

While the Xpert device has changed the landscape of TB diagnosis in resource-limited settings, the full benefit of the test can be realized only if rational evidence-based approaches towards placement decisions are adopted. Our analysis demonstrates that in Uganda, optimal placement of Xpert is dependent upon diagnostic algorithm, degree of device availability, and key health care site characteristics. When Xpert use is integrated with smear microscopy, placement of the device based on TB prevalence is superior to all other strategies regardless of the degree of Xpert rollout. This strategy is successful as higher TB prevalence improves the positive predictive value of the test. Placement of Xpert based on ART volume is second best at lower degrees of Xpert rollout, whereas placement based on poor EQA performance is equivalent to placement by TB prevalence at greater degrees of Xpert rollout. This may be explained by improved CDR with Xpert in HIV-infected individuals concentrated in high volume ART clinics, with loss of this advantage after early saturation of these clinics. At higher degrees of rollout, placement based on EQA performance capitalizes on the greater incremental sensitivity that Xpert holds over microscopy at these sites. Placement of Xpert devices at sites with poor microscopy performance addresses the known limitations of smear microscopy such as dependence on operator skill and poor diagnostic sensitivity in the setting of high HIV incidence [20–22]. When Xpert completely replaced smear microscopy in the diagnostic algorithm, the slightly lower overall CDR could be explained by the combination of test sensitivities of both smear microscopy and Xpert in the integrated diagnostic algorithm.

In our analysis the costs of initial implementation of Xpert was lowest based on prioritization of sites with greatest smear volume, which can be considered intuitive. However the cost per additional TB case diagnosed compared to smear microscopy alone was lowest when sites were prioritized by TB prevalence, a finding that highlights the need to consider the long-term implications of placement strategies.

While our study is novel in its use of multiple site level data to inform placement decisions of a new diagnostic test, there are several limitations to consider. Relative differences in CDR rates between the best and worst performing placement strategies were small (2.3%). When considering high TB burden settings such as Uganda, however, where 65,000 incident cases are estimated per year, these differences could be considered clinically significant (1500 additional cases per year). Several sites had very few observations of the EQA data which could have introduced noise in the estimate of their EQA performance. In sensitivity analysis we found that the superiority of the TB prevalence strategy at higher degrees of Xpert rollout remained stable, however the results for prioritization of sites by EQA performance did not. This underscores the need for strengthening EQA data collection and further investigation of this approach.

We did not allow for Xpert performance to vary across sites. However, given the automated platform and minimal operator hands-on time required to use the device we assumed that this variation would be minimal. We did not have site level data on HIV prevalence, which may have impacted rate of MDR-TB detection in the ART volume strategy. We did not have site-specific data to quantify the impact of reduced turnaround time for results on reduced loss to follow-up of patients. While anecdotal evidence supports such a relationship, very few studies have actually quantified it [23].

We only analyzed discrete placement strategies that have been most widely discussed in the policy and implementation literature and that are each based on a single criterion. It may be possible further elaborate upon this placement model using mathematical optimization of combined strategies as demonstrated in the case of early infant HIV diagnosis (Deo and Sohoni, forthcoming in the journal “Manufacturing and Service Operations Management”). Finally we did not include personnel, training, and infrastructural costs such as electricity necessary for Xpert implementation as these data were not readily available.

In conclusion, we found that for Uganda, placement of the Xpert device in sites prioritized by high TB prevalence was superior to other strategies based on optimization of case detection rate and implementation cost. More broadly, our work makes two important contributions. First, our analytical framework demonstrates the value of combining operational decisions regarding site selection with clinical decisions regarding diagnostic algorithm. Second, it underlines the value of using program level data (e.g. smear volume, EQA performance) to inform critical decisions and strengthen regional capacity surrounding the placement of new technology. Future areas of research should include the assessment of placement decisions on outcomes such as treatment initiation or life expectancy, the impact of point-of-care versus central laboratory placement of Xpert, and the impact of device placement on geographical patient-initiated provider-directed referral patterns.

## Supporting Information

S1 Dataset This table contains data of sputum smear microscopy workload, performance by External Quality Assessment, and cumulative ART enrollment from individual sites as obtained from the National TB Reference Laboratory (NTRL), the National TB Control Program, and the National AIDS Control Program for 2011. (PDF)
