# Supplementary material for: Optimizing Tuberculosis Case Detection through a Novel Diagnostic Device Placement Model: The Case of Uganda
Source: PLoS One. 2015 Apr 1;10(4):e0122574. doi: 10.1371/journal.pone.0122574 (PMC4382196; doi:10.1371/journal.pone.0122574)
Supplement: S1 Dataset — (PDF) [file pone.0122574.s001.pdf]

S1. Ste Level Dataset. Data from individual sites as obtained from the National TB Reference Laboratory, the National TB Control Program, and the National AIDS Control Program for 2011

Facility Name	Level	District	Smear vol	eqa % Pos smears ex	wl % Pos smears ex	eqa % Scanty smears ex	wl % Scanty smears ex	Tested	ART	eqa Pos smears recheck	eqa Scanty smears recheck	eqa Neg smears recheck	eqa HFP recheck	eqa HFN recheck	eqa LFN recheck	wl Pos	wl Scanty	wl Neg	wl %Pos	wl % Scanty
MICROBIOLOGY	NRH	KAMPALA	5309	0.15031	0.15031	0.0239	0.0239	3539	0	2	0	12	0	0	0	798	127	4384	0.15	0.024
WARD 5 & 6	NRH	KAMPALA	3119	0.17095	0.17095	0.0252	0.0252	2079	0	6	0	27	2	0	0	583	70	2466	0.187	0.022
ASSESSMENT CENTRE	NRH	KAMPALA	2134	0.27353	0.27353	0.0289	0.0289	1423	6285	8	0	22	2	0	0	582	62	1490	0.273	0.029
JINJA HOSPITAL	RRH	JINJA	1560	0.11951	0.11951	0.0227	0.0227	1040	1206	10	6	24	0	0	0	222	32	1306	0.142	0.021
FORTPORTAL R.R. HOSPITAL	RRH	KABAROLE	1719	0.1757	0.1757	0.0151	0.0151	1146	3791	12	3	20	0	0	0	334	22	1363	0.194	0.013
IDI	SP Clinic	KAMPALA	1928	0.10373	0.10373	0.0415	0.0415	1285	7529	0	0	0	0	0	0	200	80	1648	0.104	0.041
KIBOGA HOSPITAL	Hosp	KIBOGA	1811	0.07277	0.07277	0.0104	0.0104	1207	949	5	0	34	0	0	0	130	16	1665	0.072	0.009
MITYANA HOSPITAL	Hosp	MITYANA	1538	0.14927	0.14927	0	0	1025	858	10	0	26	0	0	0	246	0	1292	0.16	0
TORORO HOSPITAL	Hosp	TORORO	1691	0.14679	0.14679	0	0	1127	622	8	0	31	1	0	0	257	0	1434	0.152	0
KAMULI HOSPITAL	Hosp	KAMULI	1629	0.06602	0.06602	0.0115	0.0115	1086	646	6	5	28	0	0	0	112	13	1504	0.069	0.008
BULUBA HOSPITAL	Hosp	MAYUGE	1535	0.10764	0.10764	0.0138	0.0138	1023	558	9	3	27	0	0	0	168	17	1350	0.109	0.011
PAG	HC IV	LIRA	1565	0.12819	0.12819	0.0031	0.0031	1043	563	6	0	8	0	0	0	187	5	1373	0.119	0.003
KAWEMPE	HC IV	KAMPALA	1147	0.1539	0.1539	0.023	0.023	765	1242	4	0	37	0	0	0	227	22	898	0.198	0.019
BUGIRI HOSPITAL	Hosp	BUGIRI	1113	0.12715	0.12715	0.0097	0.0097	742	874	4	0	35	1	0	0	156	8	949	0.14	0.007
KOBOKO HC IV	HC IV	KOBOKO	1147	0.11544	0.11544	0.0046	0.0046	765	103	16	0	21	2	0	0	140	3	1004	0.122	0.003
ABER HOSPITAL	Hosp	OYAM	1128	0.12098	0.12098	0.0008	0.0008	752	928	7	0	32	1	0	0	121	1	1006	0.107	0.001
MOROTO HOSPITAL	RRH	MOROTO	1109	0.1023	0.1023	0.0016	0.0016	739	142	7	0	41	2	0	0	124	1	984	0.112	0.001
BUYINJA HC III	HC IV	NAMAYING	1182	0.03641	0.03641	0.0024	0.0024	788	292	8	0	26	0	0	0	45	3	1134	0.038	0.003
KIHIIHI HC IV	HC IV	KANUNGU	1077	0.06343	0.06343	0.0157	0.0157	718	523	3	1	26	0	0	0	74	14	989	0.069	0.013
MBARARA HOSP.MICRO	Hosp	MBARARA	1014	0.10043	0.10043	0.0086	0.0086	676	7931	15	2	13	0	0	0	112	8	894	0.11	0.008
NKOZI HOSPITAL	Hosp	MPIGI	1023	0.11159	0.11159	0.0206	0.0206	682	823	7	1	34	1	0	0	126	20	877	0.123	0.02
KITEBI H.C	HC III	KAMPALA	852	0.14123	0.14123	0.0105	0.0105	568	922	8	0	27	0	0	0	137	11	704	0.161	0.013
MAYUGE HC III	HC III	MAYUGE	956	0.08318	0.08318	0.0113	0.0113	637	128	13	0	25	2	0	0	84	7	865	0.088	0.007
NAGONGERA HC IV	HC IV	TORORO	968	0.08095	0.08095	0.0029	0.0029	645	191	7	1	32	0	0	0	78	2	888	0.081	0.002
KIWOKO HOSPITAL	Hosp	NAKASEKE	942	0.10455	0.10455	0.0165	0.0165	628	625	3	1	26	0	0	0	104	13	825	0.11	0.014
KIYUNGA HC IV	HC IV	LUUKA	966	0.01175	0.01175	0	0	644	393	5	0	44	0	0	0	11	0	955	0.011	0
NAGALAMA HOSPITAL	Hosp	MUKONO	904	0.15415	0.15415	0.0158	0.0158	603	1151	9	1	28	0	0	0	146	15	743	0.162	0.017
TASO JINJA	SP Clinic	JINJA	963	0.02783	0.02783	0.005	0.005	642	4692	1	3	35	0	0	0	28	5	930	0.029	0.005
MUTOLERE HOSPITAL	Hosp	KISORO	951	0.03144	0.03144	0	0	634	199	4	0	32	1	0	0	30	0	921	0.032	0
LYANTONDE HOSPITAL	HC IV	LYANTOND	900	0.15211	0.15211	0.0103	0.0103	600	664	10	1	20	0	0	0	148	9	743	0.164	0.01
LUWERO HC IV	HC IV	LUWERO	849	0.08562	0.08562	0.0053	0.0053	566	551	5	0	21	0	0	0	69	5	775	0.081	0.006
SOROTI RR HOSPITAL	RRH	SOROTI (40	773	0.07503	0.07503	0	0	515	2790	8	0	19	0	0	0	56	0	717	0.072	0
MASINDI HOSPITAL	Hosp	MASINDI	844	0.10155	0.10155	0	0	563	641	9	0	20	2	0	0	84	0	760	0.1	0
ADUKU HC IV	HC IV	APAC	806	0.13507	0.13507	0.0011	0.0011	537	580	9	0	30	1	0	0	117	1	688	0.145	0.001
BUDAKA HC IV	HC IV	BUDAKA	801	0.17182	0.17182	0.0069	0.0069	534	235	13	0	27	1	0	0	145	4	652	0.181	0.005
MADDU HC IV	HC IV	GOMBA	807	0.06893	0.06893	0.0023	0.0023	538	391	9	0	31	0	0	0	57	2	748	0.071	0.002
KAWOLO HOSPITAL	Hosp	BUIKWE	733	0.07774	0.07774	0.02	0.02	489	1105	11	1	27	1	0	0	63	17	653	0.086	0.023
KAYUNGA HOSPITAL	Hosp	KAYUNGA	671	0.11675	0.11675	0.0083	0.0083	447	1258	13	0	25	0	0	0	94	5	572	0.14	0.007
ST JOSEPH'S HOSP	Hosp	KITGUM	815	0.15603	0.15603	0.0118	0.0118	543	1487	5	0	28	1	0	0	121	6	688	0.148	0.007
KAZO HC IV	HC IV	KIRUHURA	772	0.0681	0.0681	0.0012	0.0012	515	12	3	0	23	1	0	0	53	1	718	0.069	0.001

TASO ENTEBBE	SP Clinic	WAKISO	763	0.07784	0.07784	0.0204	0.0204	509	3364	4	0	16	0	0	0	60	13	690	0.079	0.017
REACH OUT MBUYA	SP Clinic	KAMPALA	638	0.086	0.086	0.0356	0.0356	425	915	5	0	47	0	0	0	63	19	556	0.099	0.03
NTWETWE HC IV	HC IV	KYANKWAN	706	0.09756	0.09756	0	0	471	457	4	0	36	0	0	0	73	0	633	0.103	0
BUSOLWE HOSPITAL	Hosp	BUTALEJA	745	0.0552	0.0552	0	0	497	447	7	0	24	0	0	0	42	0	703	0.056	0
RUHOKO IV	HC IV	IBANDA	679	0.11326	0.11326	0	0	453	180	7	0	23	1	0	0	77	0	602	0.113	0
NAKASEKE HOSPITAL	Hosp	NAKASEKE	721	0.10236	0.10236	0	0	481	467	7	0	39	0	0	0	77	0	644	0.107	0
MADI OPEI HCIII	HC III	LAMWO	659	0.10892	0.10892	0.0236	0.0236	439	363	5	0	34	0	0	0	70	12	577	0.106	0.018
NANKOMA HC III	HC III	BUGIRI	668	0.12299	0.12299	0.0187	0.0187	445	321	4	0	36	0	0	0	83	9	576	0.124	0.013
BUMANYA HC III	HC III	KALIRO	708	0.04178	0.04178	0.0108	0.0108	472	262	5	2	35	1	0	0	31	8	669	0.044	0.011
KISIIZI HOSPITAL	HC III	RUKUNGIRI	700	0.10991	0.10991	0	0	467	901	6	0	25	0	0	0	80	0	620	0.114	0
SERERE HC IV	HC IV	SERERE (40	688	0.0545	0.0545	0.0027	0.0027	459	305	9	2	19	0	0	0	38	0	650	0.055	0
KIRYANDONGO HOSPITAL	Hosp	KIRYANDOI	645	0.21379	0.21379	0.0069	0.0069	430	428	15	0	25	0	0	0	149	4	492	0.231	0.006
KALISIZO HOSPITAL	Hosp	RAKAI	632	0.1841	0.1841	0.0153	0.0153	421	1146	1	2	7	0	0	0	126	9	497	0.199	0.014
KABUYANDA HC IV	HC IV	ISINGIRO	655	0.05233	0.05233	0.0028	0.0028	437	216	4	0	27	0	0	0	34	0	621	0.052	0
KAMBUGA HOSP	Hosp	KANUNGU	660	0.08677	0.08677	0.0028	0.0028	440	400	4	0	26	0	0	0	58	1	601	0.088	0.002
MAKONDO HC III	HC III	LWENGO	603	0.17003	0.17003	0.0202	0.0202	402	0	4	0	24	1	0	0	113	8	482	0.187	0.013
BUKOMERO HC IV	HC IV	KIBOGA	629	0.0708	0.0708	0.0029	0.0029	419	245	8	0	35	2	0	0	47	2	580	0.075	0.003
KAMULI M HOSPITAL	Hosp	KAMULI	595	0.11061	0.11061	0.0061	0.0061	397	581	11	3	22	0	0	0	71	3	521	0.119	0.005
ST. FRANCIS NJERU HC III	HC III	BUIKWE	597	0.11846	0.11846	0.0338	0.0338	398	1381	13	0	17	0	0	0	76	22	499	0.127	0.037
BUIKWE HOSPITAL	HC III	BUIKWE	573	0.09091	0.09091	0.0016	0.0016	382	679	8	0	32	0	0	0	53	1	519	0.092	0.002
VIRIKA HOSPITAL	Hosp	KABAROLE	593	0.14937	0.14937	0.0204	0.0204	395	2051	5	1	24	0	0	0	91	13	489	0.153	0.022
NAMUNGONA	Hosp	KAMPALA	474	0.11483	0.11483	0.0032	0.0032	316	868	7	0	38	0	0	0	61	0	413	0.129	0
BWINDI COMM. HOSPITAL	HC III	KANUNGU	545	0.11309	0.11309	0.0307	0.0307	363	378	4	1	27	0	0	0	53	16	476	0.097	0.029
ISHONGORO HCIV	HC IV	IBANDA	552	0.09804	0.09804	0.0033	0.0033	368	145	4	0	26	0	0	0	56	0	496	0.101	0
TASO TORORO	SP Clinic	TORORO	587	0.2	0.2	0.0033	0.0033	391	5203	5	1	35	2	0	0	122	2	463	0.208	0.003
NAKASONGOLA IV	HC IV	NAKASONG	572	0.09651	0.09651	0.0017	0.0017	381	192	12	0	28	1	0	0	58	1	513	0.101	0.002
KIRINYA MAIN PRISON	HC III	JINJA	598	0.00334	0.00334	0	0	399	0	2	0	20	0	0	0	2	0	596	0.003	0
KITAGATA HOSPITAL	Hosp	SHEEMA	520	0.13969	0.13969	0	0	347	849	4	0	26	0	0	0	81	0	439	0.156	0
KABERAMAIDO HC IV	HC IV	KABERAMA	556	0.125	0.125	0	0	371	739	4	0	6	0	0	0	70	0	486	0.126	0
KAPCHORWA HOSPITAL	Hosp	KAPCHORW	537	0.12326	0.12326	0.0017	0.0017	358	406	8	0	22	0	0	0	69	0	468	0.128	0
BUGOBERO HC III	HC III	MANAFA (4	528	0.07491	0.07491	0.007	0.007	352	64	4	0	26	0	0	0	41	2	485	0.078	0.004
IRUNDU HC III	HC III	BUYENDE	535	0.08803	0.08803	0.0141	0.0141	357	0	5	0	34	1	0	0	40	6	489	0.075	0.011
MATETE III	HC III	SEMBABUL	486	0.125	0.125	0.0071	0.0071	324	297	12	1	17	0	0	0	67	4	415	0.138	0.008
ANGAL HOSPITAL	Hosp	NEBBI	498	0.22321	0.22321	0	0	332	204	9	0	16	1	0	0	119	0	379	0.239	0
MOYO HOSPITAL	Hosp	MOYO	460	0.1288	0.1288	0	0	307	454	7	0	22	0	0	0	64	0	396	0.139	0
BUSESA HC IV	HC IV	IGANGA	502	0.13357	0.13357	0	0	335	0	9	0	31	1	0	0	71	0	431	0.141	0
KIGANDALO HC IV	HC IV	MAYUGE	499	0.06375	0.06375	0.0073	0.0073	333	222	6	4	22	0	0	0	35	4	460	0.07	0.008
KITYERERA HC IV	HC IV	MAYUGE	487	0.08103	0.08103	0.0018	0.0018	325	251	12	1	22	1	0	0	43	1	443	0.088	0.002
KITOVU HOSPITAL	Hosp	MASAKA	515	0.15472	0.15472	0.0208	0.0208	343	1121	9	1	20	0	0	0	77	11	427	0.15	0.021
KIBUKU HC IV	HC IV	KIBUKU	328	0.35283	0.35283	0	0	219	285	9	1	20	0	0	0	15	0	313	0.046	0
PADIBE HCIV	HC IV	LAMWO	485	0.07471	0.07471	0.0057	0.0057	323	529	5	3	32	0	0	0	36	2	447	0.074	0.004
LWAMATA	HC III	KIBOGA	499	0.04457	0.04457	0.0039	0.0039	333	0	3	1	35	0	0	0	22	2	475	0.044	0.004
SEMBABULE HC IV	HC IV	SEMBABUL	449	0.07312	0.07312	0.0059	0.0059	299	137	11	1	18	0	0	0	37	2	410	0.082	0.004
BUYENDE HC III	HC III	BUYENDE	450	0.06375	0.06375	0.002	0.002	300	0	3	0	36	1	0	0	27	1	422	0.06	0.002
ARUA R. R. HOSPITAL	RRH	ARUA	2538	0.11609	0.11609	0.0104	0.0104	1692	6231	12	2	23	0	1	0	296	25	2217	0.117	0.01

GOMBE HOSPITAL	Hosp	BUTAMBAL	1102	0.12531	0.12531	0.0008	0.0008	735	1313	11	0	19	0	1	0	147	0	955	0.133	0
MASAAFU HC III	HC III	BUSIA	716	0.07234	0.07234	0.0102	0.0102	477	941	11	0	19	0	1	0	55	7	654	0.077	0.01
KULUVA HOSPITAL	Hosp	ARUA	475	0.1	0.1	0.0098	0.0098	317	341	12	0	26	1	0	1	48	5	422	0.101	0.011
MPIGI HC IV	HC IV	MPIGI	554	0.13554	0.13554	0.0083	0.0083	369	867	11	0	25	1	1	0	79	5	470	0.143	0.009
MURCHINSON BAY	Hosp	KAMPALA	484	0.08494	0.08494	0.0116	0.0116	323	746	9	1	71	0	0	1	43	6	435	0.089	0.012
KAGADI HOSPITAL	Hosp	KIBAALE	949	0.20391	0.20391	0.0103	0.0103	633	1072	10	0	22	1	1	0	196	10	743	0.207	0.011
ABOKE HC IV	HC IV	KOLE	621	0.18429	0.18429	0.0129	0.0129	414	908	9	1	28	1	0	1	122	7	492	0.196	0.011
LACOR HOSPITAL	Hosp	GULU	1450	0.23381	0.23381	0	0	967	3656	15	2	23	0	2	0	368	0	1082	0.254	0
MUKONO HC IV	HC IV	MUKONO	1214	0.10637	0.10637	0.0149	0.0149	809	245	6	3	30	1	1	0	143	19	1052	0.118	0.016
HOIMA R.R.HOSPITAL	RRH	HOIMA	1028	0.20769	0.20769	0.0009	0.0009	685	1977	8	0	30	0	1	0	220	1	807	0.214	0.001
BRAC	SP Clinic	PADER	947	0.07223	0.07223	0.0131	0.0131	631	0	8	1	19	1	1	0	74	14	859	0.078	0.015
KAWAALA H.C	HC III	KAMPALA	574	0.12433	0.12433	0.0294	0.0294	383	1123	8	0	33	0	1	0	89	18	467	0.155	0.031
NSAMBYA HOSP	Hosp	KAMPALA	3055	0.12339	0.12339	0.0177	0.0177	2037	3868	7	0	19	0	0	1	383	53	2619	0.125	0.017
RUBAGA HOSPITAL	Hosp	KAMPALA	1849	0.17631	0.17631	0.0097	0.0097	1233	1276	7	0	24	0	1	0	326	18	1505	0.176	0.01
KAGANDO HOSPITAL	Hosp	KASESE	558	0.11809	0.11809	0.0419	0.0419	372	433	7	0	13	0	0	1	69	15	474	0.124	0.027
COMBONI HOSPITAL	Hosp	BUSHENYI	646	0.07481	0.07481	0.0076	0.0076	431	1481	7	0	24	0	0	1	46	5	595	0.071	0.008
UGANDA CARES MSK	SP Clinic	MASAKA	520	0.31863	0.31863	0.0016	0.0016	347	5090	7	0	24	0	1	0	190	1	329	0.365	0.002
AMUDAT HOSPITAL	Hosp	AMUDAT	506	0.11265	0.11265	0.0017	0.0017	337	11	13	1	29	1	2	0	55	1	450	0.109	0.002
IGANGA HOSPITAL	Hosp	IGANGA	2082	0.15904	0.15904	0.0034	0.0034	1388	1100	7	0	23	1	0	1	361	6	1715	0.173	0.003
LIRA REGIONAL REFFERAL HOS	RRH	LIRA	1720	0.15742	0.15742	0.0219	0.0219	1147	4621	6	0	9	0	1	0	275	41	1404	0.16	0.024
NYENGA HOSPITAL	Hosp	BUIKWE	600	0.11128	0.11128	0.0119	0.0119	400	636	7	0	33	1	1	0	74	8	518	0.123	0.013
ADJUMANI HOSPITAL	Hosp	ADJUMANI	446	0.21223	0.21223	0.0108	0.0108	297	504	10	1	15	0	2	0	109	5	332	0.244	0.011
GULU REG HOSP	RRH	GULU	1183	0.22977	0.22977	0	0	789	1523	6	0	23	1	1	0	275	0	908	0.232	0
KIBULI HOSPITAL	Hosp	KAMPALA	588	0.12925	0.12925	0.0051	0.0051	392	341	6	0	30	1	0	1	76	3	509	0.129	0.005
BRAC HC	SP Clinic	KITGUM	1224	0.0653	0.0653	0.0047	0.0047	816	0	10	0	25	1	2	0	78	5	1141	0.064	0.004
MUBENDE HOSPITAL	RRH	MUBENDE	1251	0.15801	0.15801	0.0098	0.0098	834	2392	4	0	25	0	0	1	210	13	1028	0.168	0.01
KAABONG HOSPITAL	Hosp	KAABONG	737	0.11617	0.11617	0.0156	0.0156	491	159	16	2	22	2	1	3	87	9	641	0.118	0.012
KIDERA HC III	HC IV	BUYENDE	713	0.0853	0.0853	0.0223	0.0223	475	269	4	0	37	0	0	1	54	10	649	0.076	0.014
KALONGO HOSPITAL	Hosp	AGAGO	564	0.13651	0.13651	0	0	376	905	4	0	14	0	0	1	76	0	488	0.135	0
KILEMBE HOSPITAL	Hosp	KASESE	536	0.13912	0.13912	0.0606	0.0606	357	1123	9	0	21	1	2	0	74	20	442	0.138	0.037
KISENYI	HC IV	KAMPALA	1248	0.17396	0.17396	0.031	0.031	832	1968	7	0	26	0	1	1	296	29	923	0.237	0.023
BRAC HC	SP Clinic	NEBBI	794	0.04709	0.04709	0	0	529	0	8	0	31	1	1	1	38	0	756	0.048	0
MATANYI HOSPITAL	Hosp	NAPAK	2073	0.16928	0.16928	0.0195	0.0195	1382	311	19	0	35	0	2	4	318	17	1738	0.153	0.008
MENGO HOSPITAL	Hosp	KAMPALA	1728	0.15567	0.15567	0.0127	0.0127	1152	2711	7	0	33	1	1	1	269	22	1437	0.156	0.013
MILD MAY CENTRE	Hosp	WAKISO	1258	0.10247	0.10247	0.0102	0.0102	839	6291	3	0	17	0	1	0	135	14	1109	0.107	0.011
KISORO HOSPITAL	Hosp	KISORO	1039	0.03707	0.03707	0	0	693	700	3	0	27	0	0	1	40	0	999	0.038	0
MALONGO HC III	HC III	MAYUGE	726	0.04901	0.04901	0	0	484	0	6	0	30	3	1	0	36	0	690	0.05	0
LUZIRA UPPER PRISON	Hosp	KAMPALA	632	0.01345	0.01345	0.0135	0.0135	421	746	4	0	36	1	1	0	9	7	616	0.014	0.011
KAMWOKYA	HC IV	KAMPALA	571	0.13154	0.13154	0.019	0.019	381	2131	4	0	16	1	1	0	77	12	482	0.135	0.021
REACH OUT- KINAWATAKA	SP Clinic	KAMPALA	630	0.06412	0.06412	0.0407	0.0407	420	1110	5	0	35	1	2	0	42	21	567	0.067	0.033
NAWAIKOKE HC III	HC III	KALIRO	672	0.02755	0.02755	0.0055	0.0055	448	132	2	0	41	0	0	1	19	3	650	0.028	0.004
KAWEMPE HOME CARE	SP Clinic	KAMPALA	374	0.11494	0.11494	0.0345	0.0345	249	416	6	1	33	0	2	2	54	12	308	0.144	0.032
KISSWA	HC III	KAMPALA	1587	0.17818	0.17818	0.0264	0.0264	1058	2216	6	0	45	1	3	0	348	37	1202	0.219	0.023
DOKOLO HC IV	HC IV	DOKOLO	874	0.14952	0.14952	0.0106	0.0106	583	874	7	0	23	2	2	1	137	9	728	0.157	0.01
RUGAZI HC IV	HC IV	RUBIRIZI	434	0.07952	0.07952	0.0099	0.0099	289	229	3	0	30	0	1	1	39	5	390	0.09	0.012

ANYEKE HCIII	HC IV	OYAM	507	0.1825	0.1825	0.0037	0.0037	338	440	8	1	20	1	3	3	92	2	413	0.181	0.004
BUTABIKA HOSPITAL	NRH	KAMPALA	1007	0.16476	0.16476	0.0229	0.0229	671	510	6	0	34	1	2	2	172	18	817	0.171	0.018
MASAKA HOSPITAL	RRH	MASAKA	1727	0.18307	0.18307	0.0222	0.0222	1151	5090	4	0	25	1	3	0	333	40	1354	0.193	0.023
LUWUNGA BARRACKS	SP Clinic	WAKISO	1019	0.07762	0.07762	0.0153	0.0153	679	0	1	0	16	0	1	0	79	9	931	0.078	0.009
KABALE HOSPITAL	RRH	KABALE	640	0.11411	0.11411	0	0	427	2412	1	0	9	0	1	0	75	0	565	0.117	0
NAMUNGALWE HC III	HC III	IGANGA	493	0.09981	0.09981	0	0	329	165	4	0	34	1	2	1	50	0	443	0.101	0
NSINZE HC IV	HC IV	NAMUTUM	469	0.03689	0.03689	0.0078	0.0078	313	207	2	0	28	0	2	0	17	4	448	0.036	0.009
APAC HOSPITAL	Hosp	APAC	1584	0.10845	0.10845	0.0054	0.0054	1056	1579	6	0	33	0	7	2	174	8	1402	0.11	0.005
KIZIBA HC III	HC III	WAKISO	614	0.02729	0.02729	0	0	409	0	1	0	28	1	2	1	17	0	597	0.028	0

Key	
Facility Name	Facility name
Level	Health care center level (NRH: National Referral Hospital, RRH: Regional Referral Hospital, Hosp: General Hospital, HC IV: Health Care Level IV, HC III: Health Care Level III, SP Clinic: HIV center)
District	District
Smear Vol	Total number smear samples performed
eqa % Pos smears ex	Proportion of smears reported as AFB positive by facility reviewed by EQA
wl % Pos smears ex	Proportion of smears reported as AFB positive by facility
eqa % Scanty smears ex	Proportion of smears reported as AFB scanty by facility as reviewed by EQA
wl % Scanty smears ex	Proportion of smears reported as AFB scanty by facility
Tested	Number of individuals tested for TB
ART	Cumulative number of patients ever enrolled on ART
eqa Pos smears recheck	Number of smears reported as AFB positive by EQA
eqa Scanty smears recheck	Number of smears reported as AFB scanty by EQA
eqa Neg smears recheck	Number of smears reported as AFB negative by EQA
eqa HFP recheck	Number of smears reported as AFB positive by facility but AFB negative by EQA
eqa HFN recheck	Number of smears reported as AFB negative by facility but AFB positive by EQA
eqa LFN recheck	Number of smears reported as AFB negative by facility but AFB scanty by EQA
wl Pos	Number of smears reported as AFB positive by facility
wl Scanty	Number of smears reported as AFB scanty by facility
wl Neg	Number of smears reported as AFB negative by facility
wl % Pos	Proportion of smears reported as AFB positive by facility
wl % Scanty	Proportion of smears reported as AFB scanty by facility

ART: Antiretroviral Therapy
EQA: External Quality
Assessment
